# Evaluation of the Effect of the Sulcus Angle and Lateral to Medial Facet Ratio of the Patellar Groove on Patella Tracking in Aging Subjects with Stable Knee Joint

**DOI:** 10.1155/2018/4396139

**Published:** 2018-05-08

**Authors:** Jianghui Qin, Dongyang Chen, Zhihong Xu, Dongquan Shi, Jin Dai, Qing Jiang

**Affiliations:** Department of Sports Medicine and Adult Reconstructive Surgery, Drum Tower Hospital, School of Medicine, Nanjing University, 321 Zhongshan Road, Nanjing, Jiangsu 210008, China

## Abstract

**Purpose:**

To determine whether the sulcus angle and the lateral to medial facet ratio correlate with patella lateral displacement and tilt in patients without patella instability.

**Methods:**

Computed tomography images of the lower limb of 64 patients without known arthropathy were collected. Three-dimensional models of the lower limb with a unified coordinate system were rebuilt by using Mimics software. The sulcus angle, lateral to medial facet ratio, lateral trochlear inclination of the patellar groove, tibial tuberosity-trochlear groove (TT-TG) distance, bisect offset index, and lateral tilt of the patella were measured. Pearson's correlation test was used to determine the relationship between the aforementioned parameters.

**Results:**

Data from 51 patients were analyzed. The sulcus angle was negatively correlated with lateral tilt inclination (*p* < 0.001, *r* = 0.8406) and positively correlated with the bisect offset index (*p* = 0.003, *r* = 0.634) and patellar tilt (*p* = 0.03, *r* = 0.551); the lateral to medial facet ratio was positively correlated with TT-TG distance (*p* = 0.003, *r* = 0.643) and bisect offset index (*p* = 0.026, *r* = 0.559).

**Conclusion:**

The sulcus angle and lateral to medial facet ratio of the patellar groove can influence patella tracking in patients with stable knee joints.

## 1. Introduction

Patellofemoral complications are a common source of patient dissatisfaction after total knee arthroplasty (TKA), and they are responsible for various surgical revisions [1]. Previous studies have indicated that proper patellar tracking could reduce the rate of patellar complications, such as subluxation and dislocation, thus decreasing the occurrence of prosthesis loosening, component wear, and soft tissue impingement [2]. In the past decades, the design concept of a prosthesis has evolved from mechanical replacement to anatomical reproduction [3]. Improvements in the prosthetic design and surgical technique have largely improved patellofemoral tracking and reduced extensor mechanism complications following TKA [4]. However, instability still accounts for up to 24% of early complications after TKA using the current design of the prosthesis [5].

Moreover, there is a persistent debate about whether the patella should be resurfaced routinely during TKA [6]. Patella resurfacing is less commonly performed in Asia and selectively performed in Europe [6]. The main consideration is the higher complication rate after resurfacing, although the clinical outcome is similar between the resurfaced and unresurfaced groups [5, 7, 8]. Improving the design of the patella trochlear groove to make a native patella-friendly component is of great clinical significance.

In order to better design of the native patella-friendly component, the effect of the trochlear outline on patella tracking in healthy knee joints should be studied. Previous studies have reported several anthropometrical parameters related to patella tracking in patients with patella instability. The geometrical characteristics of the patellar groove, especially the sulcus angle, lateral to medial facet ratio (LMFR), and lateral trochlear inclination (LTI), were related to patella maltracking [9, 10]. The bisect offset index (BOI) and lateral tilt of the patella are also characteristic parameters that indicate patellar lateral shift and inclination [11]. The tibial tuberosity-trochlear groove (TT-TG) distance is used to assess lateral displacement of the patella from the patellar tendon tibial insertion [12, 13].

In this study, we aimed to determine whether the sulcus angle and lateral to medial facet ratio correlate with patella lateral displacement and tilt in patients without patella instability. We hypothesized that the sulcus angle and LMFR correlate with patella lateral displacement and patellar tilt in patients without patella instability. Patellar lateral displacement was characterized by the bisect offset index (BOI). If our hypothesis is proven, those two parameters could affect patella tracking in patients who have undergone TKA with an unresurfaced patella; therefore, the design of a native patella-friendly component should be restored in these patients to prevent patellar complications after TKA.

## 2. Materials and Methods

The study design was approved by the ethics committee of Drum Tower Hospital, School of Medicine, Nanjing University (approval number: AF/SC-07/01.0). Sixty-four patients (30 men and 34 women aged 51 to 70 years) who underwent diagnostic computed tomography (CT) angiography of the lower limb were recruited for this study. Informed consent was obtained from all individual participants included in the study. The patients were not referred to the Orthopedic Department for an examination because they were mostly considered to have occlusion of the artery or deep vein in the lower limb due to thrombosis or atherosclerotic plaque. Accordingly, further inquiry about their history of trauma, pain, instability, swelling, and decreased range of motion of the hip and knee joints was performed to exclude the patients with any known arthropathy. The patients underwent CT in the supine position with their hip and knee joints extended. The CT images were collected, and any patient with a Kellgren-Lawrence Grading Scale score over 2 for the knee joint was excluded.

**Figure 1:**
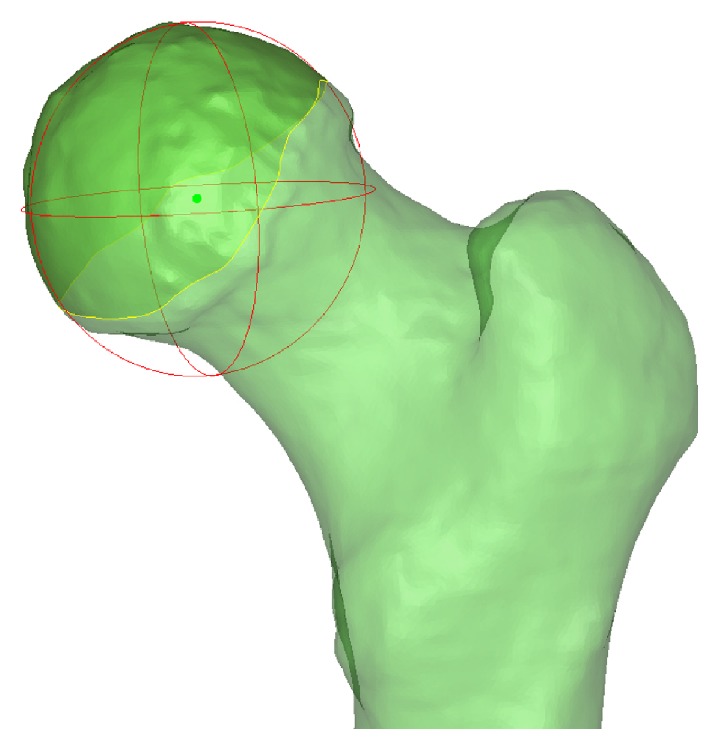
Illustration of the marked area (zone of deeper green) on the femoral head. A sphere is generated to fit the marked area, and its center (green ball) is set as the center of the femoral head.

**Figure 2:**
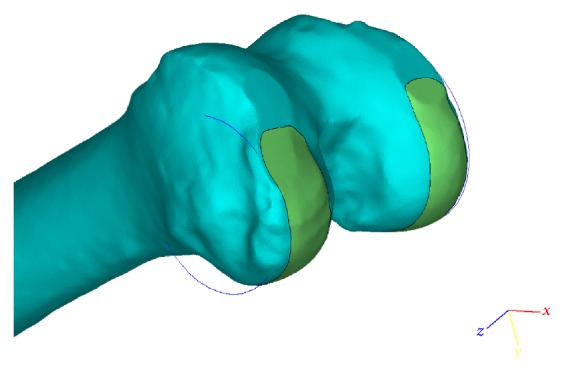
Illustration of the marked area (green color) on the femoral head. A cylinder is generated to fit the marked surface (blue circles).

CT images in DICOM format were imported into Mimics software (Materialise NV), and bone tissue of the lower limb was segmented by using the preset bone (CT) threshold of the software. Images of the segmented femur, tibia, and patella were rebuilt into a 3-dimensional model of the lower limb. Then the model was imported into a 3-matic module of Mimics software. A unified coordinate system was established according to the method invented by Leung et al. of the Chinese University of Hong Kong (method for producing knee replacement implant and implant for knee replacement, US Nonprovisional Application number 15/228,841). First, the area of the femoral head covered by cartilage was marked. A sphere was generated to fit the marked area by using the inbuilt function of Mimics software. The center of this sphere was set as the center of the femoral head (Figure 1). Next, the surface of the posterior condyle was marked. The boundary was the cartilage-covered area anterior from the height of the top of the intercondylar notch to the tip of the posterior condyle. A cylinder was generated to fit the marked surface (Figure 2). Then, the femur, tibia, hip center, and posterior condyle cylinder were merged and transferred into SolidWorks software (SolidWorks Corp.) to establish the coordinate system. The axis of the posterior condylar cylinder from the lateral to medial direction was set as the *X*-axis. The midpoint of the axis was set as the original point. The line from the original point to the center of the femoral head was set as the *Y*-axis. The *Z*-axis was set to fit the rule of a Cartesian coordinate system of 3-dimensional space.

**Figure 3:**
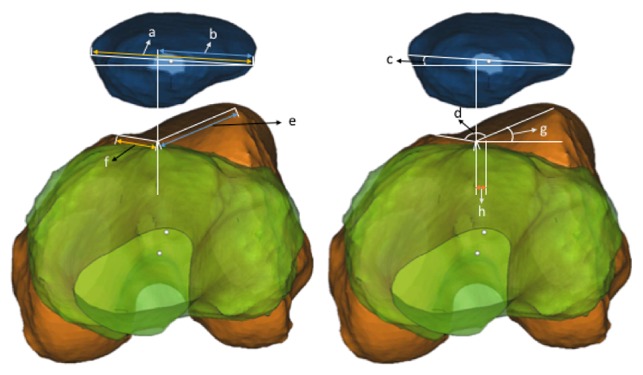
Illustration of the anatomical parameters measured. *a*: the maximum transverse diameter (MTD) of the patella. *b*: the lateral part from the projection point of the deepest point of the trochlear groove on the MTD. Bisect offset index = *b*/*a*. *c*: angle of patellar tilt. *d:* sulcus angle. *e*: length of the lateral facet. *f*: length of the medial facet. Lateral to medial facet ratio = *e*/*f*. *g*: lateral trochlear inclination. *h*: tibial tuberosity-trochlear groove distance.

The six anatomical factors that correlate with patellofemoral joint stability were measured according to the method described by Biyani et al. [14]. The lower limb was horizontal according to the plane decided by the *X*-axis and *Y*-axis and observed from the direction of *Y* axis. The measurement was performed at the highest level in which the trochlear was farthest from the plane, as determined by the *X*-axis and *Y*-axis. BOI: the maximum transverse diameter (MTD) of the patella and the projection of the deepest point of the trochlear groove on the MTD was marked. The BOI was calculated as the portion of the lateral part from the projection point on the MTD. Patellar tilt: the patellar tilt is the angle between the MTD and the plane, as determined by the *X*-axis and *Y*-axis. TT-TG distance: the TT-TG distance is the distance from the deepest point of the trochlear groove to the highest point of the tibial tuberosity. LMFR: the highest points of the lateral and medial facets were marked, and their distance to the deepest point of the trochlear groove were the lateral and medial length of the facets, respectively. The LMFR is calculated as the lateral medial length of the facets. Sulcus angle: the sulcus angle is the angle between the lateral and medial facet lines. LTI: the LTI is the angle between the lateral facet line and the plane, as determined by the *X*-axis and *Y*-axis (Figure 3).

### 2.1. Statistical Analysis

Statistical analysis was performed using Statistical Package for Social Science (SPSS) 20.0 (IBM Corp.). The skewness of all parameters was calculated to ensure that the data had a normal distribution. Differences between genders were tested by using the independent *t*-test. Pearson's correlation test was used to determine the relationship between the aforementioned parameters. The correlation coefficient (*r*) was calculated for each variable to evaluate the power of correlation. A significant difference was determined at *p* < 0.05.

## 3. Results

Fifty-one patients (25 men and 26 women) were finally analyzed in this study. Thirteen patients were excluded because they had a Kellgren-Lawrence Scale score more than 2 and previous episodes of knee pain. The average age of all patients was 63.1 years (ranges: 53–70 years in men and 51–70 years in women). There was no difference in age between sexes (*p* = 0.36, 95% confidence interval −4.60–1.70, and *t*-test). The mean value and SD of body mass index, alignment of the lower limb, MTD, length of the medial facet, and lateral facet are shown in Table 1. The mean value and SD of all six parameters including the BOI, patellar tilt, TT-TG distance, LMFR, sulcus angle, and LTI are presented in Table 2. The correlation test showed that the sulcus angle was negatively correlated with LTI (*p* < 0.001, *r* = 0.841) and positively correlated with BOI (*p* = 0.003, *r* = 0.634) and patellar tilt (*p* = 0.03, *r* = 0.551); additionally, LMFR was positively correlated with the TT-TG distance (*p* = 0.003, *r* = 0.643) and BOI (*p* = 0.026, *r* = 0.559).

## 4. Discussion

The findings of this study confirmed our hypothesis that the sulcus angle and LMFR correlate with patellar lateral displacement and tilt in patients without patellar instability. Previous studies have indicated that the sulcus angle, LMFR, LTI, BOI, patellar tilt, and TT-TG distance were related to patella maltracking in patients with patella instability. Among these factors, the LTI was found to be the strongest predictor of lateral maltracking [14]. In this study, a correlation between these parameters was found in stable knee joints. The increased sulcus angle can decrease the LTI and increase the BOI and patellar tilt. The position of the patella relative to the trochlear groove determines the lever arm of extensor mechanism and affects the efficiency of the quadriceps [15, 16]. Previous studies have demonstrated that increased LTI can affect postsurgical function of the knee joint. LTI was increased when the femoral component was internally implanted. Kawahara et al. found that internal rotation of the femoral component required higher forces of the quadriceps in deep flexion, which significantly decreased the patients' functional activities after TKA [17]. The patella engages within the trochlear groove during early flexion; thus patellar stability is critical in this stage; lateral displacement and tilt of the patellar predispose patients to patellar instability [18, 19]. In this study, the increased sulcus angle was positively correlated with the BOI and patellar tilt, which indicated that a shallow trochlear groove could result in a more laterally positioned and unstable patella. This is in agreement with previous findings that the increased patellar lateral shift and tilt were related to trochlear dysplasia [20], and a higher rate of instability was observed in the older total knee designs with shallow trochlear grooves [21, 22]. However, the anatomic design of the trochlear groove was found to be produced in more natural patellar positions, which reduced the compressive strain on the patella when the patella was unsurfaced intraoperatively [23, 24]. In conclusion, the anatomical design of the patellar groove with a proper sulcus angle and high lateral femoral flange increased patellofemoral joint congruity and decreased contact pressure on the unsurfaced patella.

An increased TT-TG distance was correlated with patellar tendinopathy and higher patellofemoral loading due to the increased lateral force vector created by the extensor mechanism on the patella [25–27]. In the present study, the LMFR was positively correlated with the TT-TG distance and BOI. This indicated that the increased LMFR could lateralize the position of the patella and increase the mechanical loading of the patellofemoral joint. This is in accordance with a previous study's finding that an abnormally high LMFR over 2.5 correlated with more severe patellofemoral degeneration [10]. Therefore, a proper LMFR is important in the design of the femoral component to prevent abnormal high stress in the patellofemoral joint.

A unified coordinate system of the lower limb based on the posterior condylar cylinder axis and midpoint of the axis and hip center was used in this study. Unlike previous anthropometric studies using the single posterior condylar axis as the reference line to measure the anatomical parameters, the coordinate system used in this study was not affected by the flexed hip or knee joint. Furthermore, because the marking area selection was preestablished and practical, the system has a high reproducibility and is not prone to observer bias.

There are limitations in this study. CT images were used in this study, so the anthropometric parameters were measured according to the bony landmarks. The exclusion of cartilage could have slightly affected the accuracy of the measurement, and the sulcus angle could have been underevaluated since the cartilage is thicker in the bottom of the groove. Besides, aging patients were recruited in this study. The deterioration of cartilage in the patellofemoral compartment could result in remolding of the subchondral bone of the patellar groove [28]. Cartilage degeneration could also affect the position of the patella in the trochlear groove. Due to the lateralized torque generated by quadriceps on the patella, decreased cartilage thickness could aggravate the lateral displacement of patella. Gender differences were not identified in this study due to the limited sample size. It is not possible to obtain a meaningful result since the sample size (*n* = 25 and 26 in male and female groups, separately) is insufficient. We will recruit more subjects in the future.

## 5. Conclusions

In this study, two anthropometric parameters of the patellar groove, sulcus angle, and lateral to medial facet ratio were found to be correlated with lateral trochlear inclination, BOI of the patella, patellar tilt, and TT-TG distance in the stable knee joint. The results indicated that the sulcus angle and lateral to medial facet ratio of the patellar groove can affect patella tracking. This is meaningful in the design of a native patella-friendly component to avoid patellar complications postoperatively, especially in the patients who received TKA with an unresurfaced patella.

## Figures and Tables

**Table 1:** Mean values and standard deviations of alignment of the lower limb (alignment), body mass index (BMI), maximum transverse diameter (MTD), length of the medial facet (MFL), and length of the lateral facet (LFL).

	Alignment (°)	BMI	MTD (mm)	MFL (mm)	LFL (mm)
Mean	178.96	25.31	44.20	11.72	21.44
SD	3.59	2.87	4.13	2.76	3.62

**Table 2:** Mean values and standard deviations of the bisect offset index (BOI), tibial tuberosity-trochlear groove (TT-TG), lateral to medial facet ratio (LMFR), and lateral trochlear inclination (LTI).

	BOI	Patella tilt (°)	TT-TG (mm)	LTI (°)	LMFR	Sulcus angle (°)
Mean	0.59	10.57	13.78	14.69	1.95	154.41
SD	0.05	5.20	3.76	4.02	0.64	8.06

## Data Availability

Details of the measurement results can be obtained by contacting the first author.
